# Retinal Vascular Density Using Optical Coherence Tomography-Angiography in Optic Neuritis

**DOI:** 10.3390/jcm12165403

**Published:** 2023-08-20

**Authors:** Paul Codron, Ines Masmoudi, Thi Ha Chau Tran

**Affiliations:** 1 Department of Ophthalmology, Amiens University Hospital, 80000 Amiens, France; codron.paul@chu-amiens.fr; 2 Department of Neurology, Amiens University Hospital, 80000 Amiens, France; masmoudi.ines@chu-amiens.fr; 3 Laboratory of Lille Neurosciences & Cognition, INSERM U1172, 59000 Lille, France

**Keywords:** optic neuritis, optical coherence tomography, angiography, vascular density

## Abstract

The aim of this study is to access the perifoveolar and peripapillary vascular density (VD) using optical coherence tomography-angiography (OCT-A) in eyes with optic neuritis (ON) and in fellow eyes, then compare that to healthy controls. Method: This is a cross-sectional study including 22 patients with unilateral ON and 20 control eyes of healthy subjects. A complete clinical examination and OCT-A were performed at least 6 months after the acute episode of optic neuritis. Vascular plexuses of the peripapillary and perifoveolar images obtained from OCT-A were used to calculate the VD in each plexus: superficial, deep, and peripapillary capillaries for each group (ON eyes, fellow eyes, healthy eyes). Results: Compared to healthy control eyes, in the peripapillary area, we found a significant decrease in VD not only in ON eyes but also in fellow eyes in average (*p* ≤ 0.05) and in the temporal sector (*p <* 0.001). In the perifoveolar area, the VD of the superficial capillary plexus is decreased in all sectors (*p* < 0.001) in ON eyes and only in the upper sector (*p* = 0.037) of fellow eyes compared to control eyes. VD correlates with ganglion cell layer (GCL) thickness in ON and in fellow eyes. Conclusion: Peripapillary vascular density is decreased in both affected eyes and fellow eyes after a unilateral episode of optic neuritis, suggesting a subclinical involvement of the disease. Further studies are needed to clarify the mechanism and clinical implications of these data.

## 1. Introduction

Optic neuritis (ON) is an inherited inflammation of the optic nerve. It was first defined by Von Graefe in 1860 [1] and further developed by Nettleship in 1884 [2]. The etiologies of ON are multiple, dominated by demyelinating pathologies such as multiple sclerosis. A diagnosis of ON is suggested on clinical examination. Magnetic resonance imaging (MRI) is required to confirm the inflammation of the optic nerve, providing information on the etiology and prognosis of the disease. A new classification of ON was established recently [3]. The diagnostic criteria are based on clinical features, allowing a diagnosis of possible optic neuritis; further paraclinical tests such as brain, orbital, and retinal imaging, together with antibody biomarker data, can lead to a diagnosis of definite optic neuritis.

Structural optical coherence tomography (OCT-B) is a retinal imaging technique that assesses the proximal part of the visual afferent pathways. Two inner retinal layers are particularly useful to study the retinal ganglion cells: the ganglion cell layer (GCL) corresponds to their bodies and the retinal nerve fiber layer (RNFL) to their axons. In the acute phase of optic neuritis, papilledema may be present in 30% of cases, leading to an increase in the RNFL thickness compared to the healthy eye [4], and the GCL is not impaired during this phase [5]. A significant loss of GCL occurs after 4 weeks, while RNFL thinning is observed significantly three months after the initial episode. Since initial papilledema may mask the RNFL thinning on OCT at the acute phase, GCL may therefore be an earlier biomarker than RNFL for the ganglion cell loss during the follow-up of optic neuritis [6,7]. Regarding the fellow eye, Behbehani et al. [7] did not demonstrate significant thinning of GCL and RNFL in the fellow eye of patients with an acute episode of ON and without a history of multiple sclerosis (MS). Akaishi et al. in 2017 [8] found thinning of the fellow eye in patients with MS and AQP4 neuromyelitis optica but not in idiopathic optic neuritis or in anti-MOG (myelin oligodendrocyte glycoprotein) autoantibody optic neuritis.

In MS patients, previous publications on OCT-A reported conflicting results, depending on the history of optic neuritis, the studied area (peripapillary, macular), the protocols and the devices used. Spain et al. showed a decrease in peripapillary vascular flow in MS patients with and without a history of ON [9]. Feucht et al. found a decrease in macular vascular density (VD) only in MS patients with ON [10]. Wang et al. demonstrated decreased peripapillary VD in MS patients with ON [11]. However, these authors did not find any vascular density damage in MS patients without ON [10,11]. More recently, Bostan et al. found an impairment of perifoveolar vascular density in MS patients without ON [12].

There is a need to clarify the retinal microvascular structure involvement in patients with optic neuritis and without neurological history by separating the ON eye from the fellow eye. Lee et al. first studied the microvascular structure of the ON eye and the fellow eye using OCT-A Cirrus (Carl Zeiss Meditec, AG, Jena, Germany) in patients without any neurological history and found peripapillary and perifoveolar microvascular damage in the fellow eyes [13]. The aim of the study is to evaluate the macular and peripapillary microvascular structures in optic neuritis patients, in both the ON-eye and in the fellow eye, at least 6 months after the first attack episode of unilateral ON.

## 2. Materials and Methods

### 2.1. Design

This is a single-center, prospective, cross-sectional study that was conducted in accordance with the Declaration of Helsinki. Patients were included consecutively between May 2019 and May 2021 in the department. IRB approval was obtained (local ethic approval number: PI2022_843_0128).

#### 2.1.1. Inclusion Criteria

##### Patients

Twenty-two patients who suffered of first attack of unilateral ON, aged from 18 to 55 years, with an OCT-A examination performed at least 6 months later after the acute episode were included. The diagnosis of ON was based on these criteria: (1): decreased visual acuity, pain on mobilization of the globe, relative afferent pupillary defect; (2) presence of a central or centrocecal scotoma in the visual field test; and (3) contrast enhancement on the T2-FLAIR sequence of the inflamed optic nerve on MRI.

##### Controls

Twenty age- and gender-matched controls were included. All controls had normal ophthalmological examinations. Only the right eye of the controls was used for the study.

Visual acuity was measured in all participants using the Monoyer scale, which was converted to log MAR for statistical purposes.

#### 2.1.2. Exclusion Criteria

The exclusion criteria were non-inflammatory optic neuropathy (glaucomatous optic neuropathy, ischemic optic neuropathy); retinal diseases (diabetes, vascular abnormalities, retinal vasculitis); history of neurological disease or diagnosis of MS prior to the first episode of ON; known history of bilateral optic neuritis; significant medial opacity; and myopia < −6D.

### 2.2. Procedure

Complete clinical examinations, OCT-B and OCT-A, were performed in both patients and controls. Visual fields and MRI were performed only in the patients’ group.

#### 2.2.1. OCT-B

Structural OCT (Spectralis™ II V6.12.4.0 SD, Heidelberg Engineering™, Heidelberg, Germany) was performed at least 6 months after the first acute episode using the RNFL and GCL protocols. We chose the 6 month interval for all patients in order to avoid bias related to papilledema in the acute phase. The analysis of the global RNFL and the GCL thickness and those of the 4 sectors (superior, inferior, temporal, and nasal) were generated by the HEYEX V1.10.4.0 software (Heidelberg Engineering™, Heidelberg, Germany).

#### 2.2.2. OCT-A

Perifoveolar and peripapillary vascular analyses were obtained from the OCT-A module (Spectralis™ II V6.12.4.0 SD, Heidelberg Engineering™, Heidelberg, Germany). Each patient received a peripapillary and perifoveolar scan in both eyes. The eye-tracker function was activated during the acquisition time. We used a 15° × 15° square for the perifoveolar area and a 20° × 20° for the peripapillary area using the high-resolution template (5.7 µm/pixel). Only scans with a signal quality >25 without misalignment, motion, or blink artefacts were selected for analysis.

The segmentation of the different plexuses was performed automatically by the HEYEX software in order to analyze the superficial vascular plexus (SVP) and the deep capillary plexus (DCP) for the perifoveolar area and the capillary network for the peripapillary area.

### 2.3. Vascular Density

The measurement of vascular density was calculated manually using the previously published EA-Tool OCT-A software, which was provided gratefully by the University of Erlangen in Germany. This software was coded in MATLAB. It is independent from the Spectralis device and has been shown to be reliable and reproducible [14]. The OCT-A scans of the SVP and DCP of the perifoveolar area were extracted in JPEG format from the Spectralis device, then exported into the application and analyzed separately to calculate the VD. A binary image was generated from an OCT-A scan of the studied plexuses, using Otsu’s algorithm, in which each vessel pixel is white, and each tissue pixel is black [15]. A first circle was drawn with a diameter of 0.8 mm covering the FAZ, and then a second circle with a diameter of 4 mm with the FAZ as its center was drawn and divided into 4 sectors at a 90° angle (Figure S1). EA-Tool calculated the percentage of “white area” in the “total area” of the region of interest, called vessel density. Foveolar avascular zones (FAZs) of the SVP and DCP were calculated manually. The OCT-A scan of the whole peripapillary vascular plexus was also extracted to calculate peripapillary VD.

### 2.4. Statistical Analysis

Analyses were performed using IBM SPSS 27 software. The results were presented as median [Q1; Q3], where Q1 and Q3 are the first and third quartiles, respectively. To study the relationship between two groups of a qualitative variable and, we used a chi-squared (χ^2^) test. The non-parametric Kruskal–Wallis test was used to study the relationship between qualitative and quantitative variables. A *p* value of <0.05 is considered significant.

## 3. Results

### 3.1. Demographic Characteristics of the Study Population

Twenty-two patients (22 ON eyes, 22 fellow eyes) and 20 control eyes were included. The characteristics of the patients and controls are summarized in Table 1.

In the patient group (fourteen females/eight males; n = 22), the median age was 36.5 years [from 25 to 49]. Baseline visual acuity was 0.4 (0.1–1) log MAR for ON eyes and 0 log MAR for fellow eyes. They were all treated with 1 g intravenous methylprednisone during their acute episode. Visual acuity improved to 0 log MAR at 6 months. The mean follow-up time was 17 months (Q1–Q3: [12; 36.5]). At the end of the acute episode, eleven patients were diagnosed with multiple sclerosis, two with neuromyelitis optica, and one with acute disseminated encephalomyelitis (ADEM).

In the control group, 20 participants were included (n = 20, thirteen females/seven males), and the median age was 30 years [22–43.75].

### 3.2. Vessel Density on OCT-A

The results of the vessel density between the three groups (eyes with ON, fellow eyes, and healthy eyes) are summarized in Table 2.

#### 3.2.1. Radial Peripapillary Capillary (RPC) Density

There was no difference in vessel density (VD) between eyes with ON vs. fellow eyes. However, VD of the RPC was significantly reduced in the average (*p* = 0.008), in the nasal (*p* = 0.025), and temporal sectors (*p* < 0.001) of ON eyes compared to the control eyes. The VD of the RPC was significantly reduced in the average (*p* = 0.050) in the temporal sector (*p* < 0.001), and there is a trend of thinning in the nasal sector (*p* = 0.07) of fellow eyes compared to control eyes.

#### 3.2.2. Perifoveolar Vessel Density

- Superficial Vascular Plexus (SVP)

In eyes with ON, the vessel density of the superficial capillary plexus was reduced in the average (*p* = 0.034,) in the nasal (*p* = 0.009) and temporal (*p* = 0.012) sectors compared to fellow eyes. It was significantly reduced in all four sectors (*p* < 0.001), compared to control eyes. In fellow eyes, vessel density was significantly reduced in the superior sector (*p* = 0.037) compared to control eyes.

- Deep Capillary Plexus (DCP)

We did not find any significant difference in DCP analysis between the three groups.

### 3.3. Structural Analysis at OCT-B

Table 3 shows the comparison of the retinal layer thickness between the three groups.

#### 3.3.1. Retinal Nerve Fiber Layer (RNFL)

In eyes with ON, RNFL thickness was significantly reduced in the average (*p* = 0.004), in the superior (*p* = 0.038), inferior (*p* = 0.024), and temporal (*p* = 0.006) sectors compared to fellow eyes. It was also reduced in the average (*p* < 0.001), in the superior (*p* = 0.001), nasal (*p* = 0.001), inferior (*p* = 0.003), and temporal (*p* < 0.001) sectors compared to control eyes.

There was no difference in RNFL thickness between fellow eyes of patients and control eyes.

#### 3.3.2. Ganglion Cell Layer (GCL)

In eyes with ON, GCL thickness was significantly reduced in the average (*p* < 0.001) as well as in the superior (*p* < 0.001), temporal (*p* < 0.001), nasal (*p* = 0.003), and inferior (*p* = 0.001) sectors compared to fellow eyes. It was also significantly reduced in all four sectors (*p* > 0.001) compared to control eyes. In the fellow eyes, the GCL thickness was significantly reduced in the nasal sector (*p* = 0.015), compared to the control eyes.

### 3.4. Relationship between Retinal Structure and Vascularization in Eyes with Optic Neuritis

In the ON eyes, there is a relationship between the vessel density of SVP and the RNFL thinning in the superior and inferior sectors (superior, *p* = 0.05; inferior, *p* = 0.035). Similarly, the VD of the SVP was related to the loss of GCL thickness in the corresponding sectors: superior, inferior, and nasal (superior, *p* = 0.007; inferior, *p* = 0.034; nasal, *p* = 0.007).

In the fellow eyes, we did not find any correlation between vessel density and RNFL. There is a correlation between SVP vessel density and GCL thickness in the corresponding superior and temporal sectors (superior = 0.04; temporal, *p* = 0.004).

We did not find any relationship between the OCT-B and OCT-A parameters and the duration of disease, the MS diagnosis after the initial episode, or visual acuity.

## 4. Discussion

OCT-A and OCT-B are non-invasive, fast, reproducible, and highly accurate examinations. OCT-A allows direct access to retinal perfusion, which is supposed to reflect cerebral perfusion in demyelinating pathologies. Therefore, these techniques have potential applications in research on neurodegeneration.

Our study showed that in the eyes with optic neuritis, there is an impairment of the retinal microvascular structure in the peripapillary and perifoveolar areas compared to the control eyes. In the fellow eyes, we found a decrease in peripapillary VD as well as a decrease in SVP VD in the upper sector of perifoveolar area, compared to the control eyes. In eyes with ON, we did not observe any difference in VD at the peripapillary area, but there was a decrease in the SVP VD of the perifoveolar area compared to that of the fellow eyes.

Our study showed a decrease in VD in the eyes with ON, which is consistent with the literature [16,17]. We also demonstrated retinal microvascular damage in the fellow eyes of patients who suffered of first unilateral attack of ON and who had no neurological history. Similarly, Lee et al. reported retinal vascular damage in the contralateral eyes, in the upper sector of the peripapillary area, and in the average perifoveolar area. In our study using Heidelberg OCT-A, we found a decrease in VD in these two areas: in the temporal sector of the peripapillary area and in the superior sector of the perifoveolar area. Differences in sectors involvement in these peripapillary and perifoveolar areas might be related to the use of different devices, since Heidelberg OCT-A has an integrated eye-tracker, and/or differences in the study population. The study of Lee et al. included more patients with neuromyelitis optica (NMOSD) who might have greater vascular involvement than patients with multiple sclerosis [18], whereas half (11/22) of our patients had MS and none had NMOSD.

The decrease in vessel density in the ON eyes and in the fellow eyes could be explained by two hypotheses. In the first hypothesis, there is a reduction in metabolic demand resulting from axonal and neuronal loss (RNFL and GCL) [10], leading to a regression of the vessels of the superficial plexus, which vascularized the axons and bodies of the retinal ganglion cells (RNFL and CGL) [19]. This hypothesis is consistent with other reports showing a decrease of cerebral perfusion in areas of apparently healthy white substance in MS [20]. This hypoperfusion would be due to impairment in astrocyte metabolism, in particular poor K+ absorption leading to poor arteriolar dilatation [21]. In the second hypothesis, a rarefaction of these vessels is attributed to retinal vessel inflammation during an episode of optic neuritis, which leads to axonal and neuronal loss. This hypothesis is supported by our results and Lee et al.’s report [13], showing that deep capillary plexus, which vascularizes the outer layers of the retina, is unaffected in optic neuritis. Lanzillo et al. [17] demonstrated similarly a reduction of retinal vessel density in MS patients eyes even without an episode of ON, suggesting a primary vascular attack concomitant with demyelination. This second hypothesis would explain the decrease in peripapillary VD observed in the fellow eyes in the remission phase of optic neuritis attack in our study (>6 months). The primary vascular impairment would occur before or concomitantly with the discovery of the demyelinating disease. The vascular perfusion might play a role in the pathophysiology of demyelinating diseases, which is revealed using different techniques such as perfusion MRI and more recently OCT-A [20,22,23].

The thinning of RNFL in the ON-eyes compared to the control group found in our study has been reported previously [7,24,25]. This damage can be explained by retrograde degeneration after an episode of ON, with axonal loss leading to ganglion cell loss. Furthermore, in the fellow eyes, we found a significant thinning of the GCL layer in the nasal sector compared to the control eyes, while there was no difference in RNFL thickness between the two groups. According to a meta-analysis [24], the GCL layer decreases in MS patients even without ON. The GCL layer seems to be more sensitive than the RNFL in detecting structural damage after an episode of optic neuritis [7,26], and the thinning of the GCL seems to reflect demyelinating disease activity and neurodegeneration [27].

Our study has the strength of a rigorous selection of patients according to clinical and MRI criteria, leading to a homogenous study population. In addition, the patients in this study did not have any neurological disease at the time of their first unilateral attack of optic neuritis, therefore, were not exposed to a possible prior loss due to a demyelinating disease. It also has the strength of measurement reliability using the integrated eye tracker of the OCT-A device. However, we acknowledge several limits: (1) a Heidelberg OCT-A database is lacking from the device, which is compensated by the constitution of a control group; and (2) the size of the study population does not allow subgroup analyses of etiology (MS, anti-AQP4, anti-MOG). All patients were assessed in the remission phase of the disease (>6 months), so it is difficult to know when the vascular damage occurred.

Our study is among the firsts to show latent retinal microvascular damage in the fellow eyes, which is supposed to be normal, in patients who suffered their first attack of unilateral ON without neurological disease history. We do not know when the microvascular alteration occurred—at the onset of ON or 6 months later during the remission phase. A decrease in VD in these fellow eyes suggests a subclinical microvascular impairment of an underlying inflammatory pathology. These eyes would be more likely to develop a demyelinating disease in the course of history. Indeed, a decrease in perfusion is part of the pathophysiology of MS [20,22,23], and microvascular involvement has been previously reported in MS patients without a history of ON [12]. The clinical impact and pathophysiological mechanism of retinal vascular alteration need to be elucidated in order to know if retinal perfusion could have a place in the follow-up of patients with inflammatory neurological diseases. Indeed, some studies find an improvement in retinal VD during clinical stabilization in the follow-up of MS patients [28].

## 5. Conclusions

The results of our study demonstrated that structural and vascular damages were found not only in the ON-eyes but also in the fellow eyes. This alteration might result from a pre-existing background disease that was not discovered during the first episode of optic neuritis. Further studies are needed to clarify the relationship between disease progression and retinal microvascular damage and to determine the place of vessel density measurement in the monitoring and treatment of inflammatory diseases of the central nervous system.

## Figures and Tables

**Table 1:** Clinical and demographic characteristics of patients with optic neuritis and controls.

	Patients			
	Eye with Optic Neuritis (n = 22)	Contralateral Eye (n = 22)	Controls (n = 20)	*p* Value
Variables				
Gender, Female/Male	14/8		13/7	0.927
Age, years, mean [Min–Max]	36.5 [25–49]		30.00 [22–43.75]	0.217
Spherical equivalent (diopters)	0 [−1.25; 0]		0 [−0.94; 0.50]	0.364
Initial BCVA (log Mar)	0.4 [0.1; 1]	0 [0; 0]	0 [0; 0]	<0.001
BCVA at 6 months (log Mar)	0 [0; 0.2]	0 [0; 0]	0 [0; 0]	0.001
Follow-up duration (months)	17 [12; 36.5]			
MS diagnosis after ON (%)	11 (50%)		0	

BCVA = best-corrected visual acuity; ON = optic neuritis.

**Table 2:** Comparison of vascular density between the superficial, deep, and peripapillary plexus in the three groups (ON eyes, fellow eyes, and control eyes).

	ON Eyes (n = 22)	Fellow Eyes (n = 22)	ON vs. Fellow Eye	Controls (n = 20)	ON vs. Control Eye	Fellow Eye vs. Control Eyes
			(*p*)		(*p*)	(*p*)
Perifoveolar area						
SVP %						
Average	22.11 [16.38; 24.30]	26.90 [22.55; 29.28]	0.034	29.39 [27.98; 31.29]	<0.001	0.069
Upper	23.00 [17.48; 27.05]	26.85 [22.50; 30.25]	0.163	31.60 [29.28; 32.73]	<0.001	0.037
Nasal	20.25 [15.18; 23.88]	25.95 [22.53; 28.83]	0.009	28.25 [26.48; 29.85]	<0.001	0.218
Inferior	22.95 [16.23; 26.65]	27.95 [22.48; 29.73]	0.062	29.50 [28.10; 32.35]	<0.001	0.112
Temporal	21.70 [18.23; 24.63]	25.60 [22.20; 29.60]	0.012	28.5 [26.98; 30.18]	<0.001	0.131
DCP, %						
Average	20.96 [17.83; 2320]	22.29 [20.23; 24.18]	/	22.64 [20.00; 24.31]	0.134	/
Upper	21.95 [15.25; 25.03]	24.00 [21.50; 25.88]	/	22.65 [18.73; 25.70]	0.260	/
Nasal	20.25 [15.90; 22.48]	21.80 [18.60; 23.83]	/	21.25 [20.00; 23.03]	0.167	/
Inferior	20.65 [18.65; 23.35]	21.80 [20.20; 24.28]	/	22.80 [20.20; 24.73]	0.192	/
Temporal	19.95 [18.10;23.28]	21.55 [19.38; 23.00]	/	22.45 [19.70; 24.38]	0.205	/
Peripapillary area (radial peripapillary capillary) %						
Average	29.24 [25.29; 31.12]	30.05 [25.93; 33.37]	1.000	33.06 [32.08; 34.44]	0.008	0.050
Upper	28.60 [25.30; 34.80]	29.60 [26.63; 32.45]	/	30.85 [29.30; 32.25]	0.597	/
Nasal	31.70 [27.93; 34.93]	30.55 [28.73; 34.75]	1	34.25 [32.58; 35.65]	0.025	0.072
Inferior	30.20 [23.58; 33.35]	29.75 [26.88; 33.98]	/	32.25 [29.18; 33.75]	0.392	/
Temporal	25.70 [18.28; 31.38]	29.90 [22.85; 31.48]	1.000	34.65 [32.28; 37.33]	<0.001	<0.001

SVP = superficial vascular plexus, DCP = deep capillary plexus, RPC (radial peripapillary capillary) peripapillary capillary network. The values are: median [Q1; Q3], where Q1 and Q3 are the first and third quartiles, respectively. (*p*) value was calculated by a Kruskal–Wallis test with Bonferroni correction.

**Table 3:** Comparison of retinal thickness between controls, ON eyes, and fellow eyes.

	ON Eyes (n = 22)	Fellow Eyes (n = 22)	ON Eyes vs. Fellow Eyes (*p*)	Controls (n = 20)	ON Eyes vs. Control Eyes (*p*)	Fellow Eyes vs. Control Eyes (*p*)
RNFL thickness (μm)						
Average	84.50 [70.75; 93.00]	96.50 [90.50; 103.75]	0.004	100.00 [97.00; 103.00]	<0.001	0.715
Upper	111.00 [83.75; 121.25]	119.00 [116.25; 122.00]	0.038	125.50 [107.00; 132.25]	0.001	1.000
Nasal	54.50 [49.00; 69.00]	63.00 [54.75; 79.50]	0.273	71.00 [66.75; 77.00]	0.001	0.181
Inferior	105.00 [81.75; 120.25]	128.50 [107.50; 135.75]	0.024	128.50 [119.50; 133.00]	0.003	1.000
Temporal	51.50 [40.50; 66.00]	71.00 [60.75; 81.00]	0.006	75.50 [71.25; 81.00]	<0.001	0.656
GCL thickness (μm)						
Average	34.13 [26.94; 42.19]	50.25 [46.69; 52.88]	0.001	52.50 [51.25; 54.19]	<0.001	0.136
Upper	37.00 [30.00; 43.50]	51.00 [48.50; 55.25]	<0.001	54.50 [52.00; 57.00]	<0.001	0.261
Nasal	34.50 [24.75; 42.25]	50.00 [44.25; 51.50]	0.003	53.50 [52.00; 55.75]	<0.001	0.015
Inferior	36.00 [31.50; 43.50]	50.50 [48.50; 54.25]	0.001	53.50 [52.00; 55.75]	<0.001	0.209
Temporal	31.50 [23.00; 38.25]	47.50 [43.75; 50.25]	<0.001	48.50 [47.00; 52.75]	<0.001	0.376

SE = standard error; GCL = ganglion cell layer; RNFL = retinal nerve fiber layer. The values are: median [Q1; Q3], where Q1 and Q3 are the first and third quartiles, respectively. (*p*) values are calculated by a Kruskal–Wallis test with Bonferroni correction.

## Data Availability

Data are available upon request.

## Supplementary Materials

The following supporting information can be downloaded at: https://www.mdpi.com/article/10.3390/jcm12165403/s1. Figure S1: Calculation of vessel density of the Superficial Vascular Plexus and Deep Vascular Plexus in the perifoveolar area.
