# Supplementary material for: Retinal Vascular Density Using Optical Coherence Tomography-Angiography in Optic Neuritis
Source: J Clin Med. 2023 Aug 20;12(16):5403. doi: 10.3390/jcm12165403 (PMC10455229; doi:10.3390/jcm12165403)
Supplement: Supplementary file 1 [file jcm-12-05403-s001.zip › jcm-2525821-supplementary.pdf]

Supplemental data

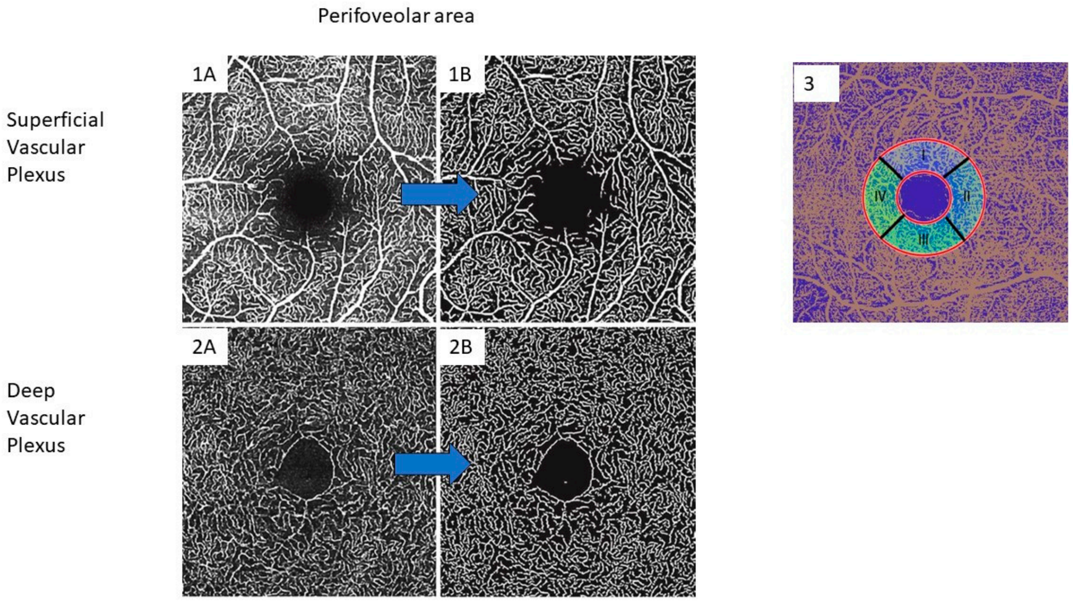

Figure S1. Calculation of the Superficial Vascular Plexus and Deep Vascular Plexus vascular density in the perifoveolar area. The OCT-A scans of the SVP (1A) and DCP (2A) of the perifoveolar area were extracted in JPEG format from Spectralis device, then exported into the application to calculate of VD. The image was binarized using Otsu's algorithm (1B and 2B), each vessel pixel is white, and each tissue pixel is black. A first circle was drawn with a diameter of 0.8mm covering the FAZ and then a second circle with a diameter of 4mm with the FAZ as its center was drawn and divided into 4 sectors of 90° angle (3). EA-Tool calculated the percentage of "white area" in the "total area" of the region of interest, called vessel density.
